# Leser-Trélat syndrome in patients affected by six multiple metachronous primitive cancers

**DOI:** 10.1186/1756-8722-3-2

**Published:** 2010-01-11

**Authors:** Giovanni Ponti, Gabriele Luppi, Lorena Losi, Alberto Giannetti, Stefania Seidenari

**Affiliations:** 1 Department of Oncology and Haematology, University of Modena and Reggio Emilia, Modena, Italy; 2 Section of Pathological Anatomy, University of Modena and Reggio Emilia, Modena, Italy; 3 Division of Dermatology, University of Modena and Reggio Emilia, Modena, Italy

## Abstract

Leser-Trélat syndrome is characterized by the eruptive appearance of multiple seborrheic keratoses in association with underlying malignant disease. Usually, the sign of Leser-Trélat is associated with adenocarcinoma, most frequently of the colon, breast, or stomach, but also of the lung, kidney, liver, and pancreas. Herein, we present a case that we believe is the first report of the sign of Leser-Trélat in association with occult gastric adenocarcinoma and the anamnestic oncologic history of five other multiple primitive cancers. Epidermal growth factor receptor (EGFR) immunohistochemical expression analysis of multiple seborrheic keratoses revealed an intense membranous staining in the basal keratinocytes and in all the upper epidermal layers. Patients with the sign of Leser-Trélat should undergo a diagnostic screening programme for malignant disease along with patients with known Leser-Trélat syndrome who present with a recent acute and florid eruption of their seborrheic keratoses. We propose the importance of combining the molecular features of multiple seborrheic keratoses with EGFR immunohistochemistry analyses to determine the likelihood of Leser-Trélat syndrome and the consequent high risk of underlying multiple visceral malignancies.

## Background

Skin manifestations are frequently observed in many internal malignancies. Cutaneous lesions, including benign neoplasms or pigmented lesions, can appear in the context of specific genodermatosis [1,2], lentiginosis [3], or paraneoplastic syndrome [4] representing a crucial cutaneous marker of internal malignancy in both the sporadic and genetic setting. Several familial cancer syndromes have additional malignant or non-malignant cutaneous features that form part of the clinical diagnostic criteria constituting a critical tool for clinical diagnosis.

Several cutaneous paraneoplastic syndromes may be associated with underlying tumours. They include musculoskeletal disorders (clubbing, hypertrophic osteoarthropathy, dermatomyositis, and multicentric reticulohistiocytosis), reactive erythemas (erythema gyratum repens and necrolytic migratory erythema), vascular dermatoses (Trousseau's syndrome), papulosquamous disorders (acanthosis nigricans, tripe palms, palmar hyperkeratosis, acquired ichthyosis, pityriasis rotunda, Bazex's syndrome, florid cutaneous papillomatosis and extramammary Paget's disease), and disorders of hair growth (hypertrichosis lanuginosa acquisita) (Table 1) [4]. The clinical manifestations of these dermatoses may precede, coincide with, or follow the diagnosis of cancer. Most paraneoplastic dermatoses disappear when the primary tumour is removed and reappear in the case of recurrence or metastases of the cancer.

**Table 1:** Cutaneous manifestations of internal malignancies

Cutaneous feature	Clinical findings	Associated malignancy
**Acanthosis nigricans (malignant)**	Velvety hyperpigmented thickening extending beyond the flexures and neck to involve lips, palms	Intra-abdominal adenocarcinoma, lung carcinoma, lymphoreticular malignancies

**Acrokeratosis paraneoplastica (Bazex's syndrome)**	Psoriasiform dermatitis with nail dystrophy affecting hands, feet, ears, nose	Squamous cell carcinoma of upper aerodigestive tract

**Erythema gyratum repens**	Wood grain pattern annular, scaling erythema	Lung carcinoma

**Necrolytic migratory erythema**	Eroded erythematous annular polycyclic eruption affecting intertriginous areas	Glucagonoma

**Sweet syndrome**	Plum coloured nodules affecting head, neck and dorsae, hands	Can be associated with leukaemia, lymphoma, multiple myeloma

**Pyoderma gangrenosum especially bullous variant**	Painful inflammatory ulcers with raised violaceous edge and overhanging borders; associated pathergy	Can be associated with leukaemia, lymphoma, multiple myeloma

**Paraneoplastic pemphigus**	Bullous, erosive mucosal +/- cutaneous eruption	Haematologic malignancies, thymoma

**Necrobiotic xanthogranuloma**	Purpuric yellow plaques in periorbital and flexural areas	Monoclonal gammopathy/multiple myeloma

**Diffuse plane xanthomas**	Yellow-orange macules and plaques	Monoclonal gammopathy/multiple myeloma

**Scleromyxoedema**	Scleroderma-like thickening of skin associated with coloured skin or erythematous papular infiltrate	Monoclonal gammopathy/multiple myeloma

**Primary systemic amyloidosis**	Macroglossia, purpura especially periorbital and infiltrated papules	Monoclonal gammopathy/multiple myeloma

The sign of Leser-Trélat is characterized by the eruptive appearance of multiple seborrheic keratoses (SK) in association with underlying malignant disease. Two European surgeons, Edmund Leser and Ulisse Trélat, were the first to describe the sign, and although they gave no mention of the presence of seborrheic keratoses reporting a specific association with angiomas - that probably does not exist - their names continue to be linked to a different paraneoplastic association [5]. In 1900, a subsequent report clearly defined the association of seborrheic keratosis and cancer [6]. Usually, the sign of Leser-Trélat is associated with adenocarcinoma, most frequently of the colon, breast, or stomach, but also of the kidney, liver, and pancreas, among others [7,8].

Endogenous mediators of hyperproliferative skin disease such as epidermal growth factor (EGF), transforming growth factor-alpha (TGF-α), or amphiregulin that may function in a localized autocrine manner or be found in the circulation affecting the epidermis more diffusely, may induce some seborrheic keratoses [9,10].

Herein, we present a case that we believe is the first report of Leser-Trélat syndrome in association with occult gastric adenocarcinoma and the anamnestic oncologic history of five other multiple primitive cancers.

## Case presentation

**Figure 1:**
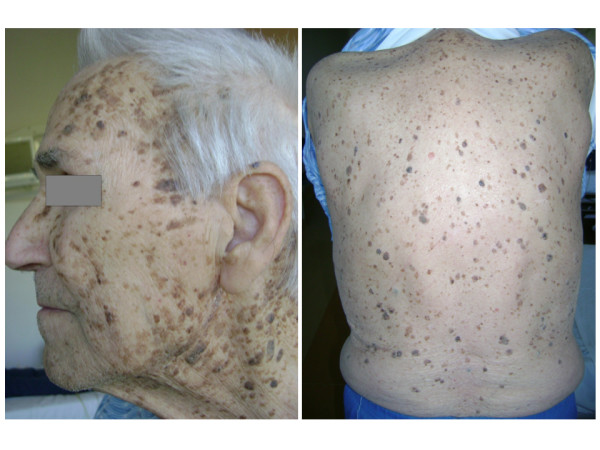
**Clinical presentation of innumerable seborrheic keratoses concentrated over the face, neck, back, and chest**.

**Figure 2:**
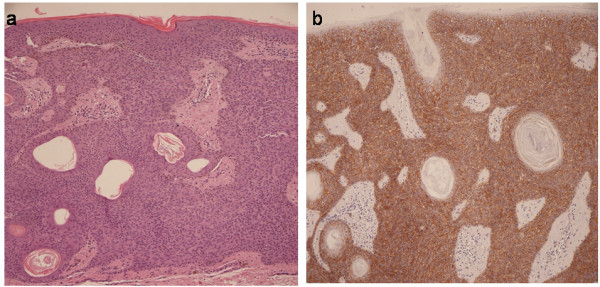
**(a) Histopathologic features and (b) immunohistochemical expression of EGFR in specimen from back lesion showing intense membranous staining of EGFR in the basal keratinocytes and throughout the upper epidermal layer**.

A 83-year-old man was admitted to our hospital in August 2009 with a recent history of dyspnea, cough and dyspepsia. His medical history was characterized by sigmoidectomy and ileal-cecal resection for two synchronous adenocarcinomas (T1N0M0) in August 2008; localized adenocarcinoma of the prostate diagnosed in 2007 treated with hormonotherapy and radiotherapy with radical intent; cutaneous basal cell carcinoma and squamous cell carcinoma removed about 10 years before. For the past 25 years, he had noted multiple asymptomatic lesions, diagnosed as multiple seborrheic keratosis, of the face and trunk and these had recently increased in size and number with generalized pruritis. On admission, dermatologic examination noted florid eruption of innumerable keratotic lesions of the face, neck and thorax, ranging in size from 3 to 15 mm (Figure 1). Histopathological examination of a surgical skin biopsy confirmed the clinical diagnosis of seborrheic keratosis and was used for EGFR immunohistochemical analysis (Figure 2). Immunoperoxidase staining using diaminobenzidine as chromogen was run with the Benchmark XT automatic staining system (Ventana Medical Systems Inc, Strasbourg, France). Mouse monoclonal antibody anti-EGFR (clone 31G7; Ventana, Strasbourg, France) was used at a dilution of 1:100.

In July 2009, computed tomography (CT) of the chest and abdomen revealed a 2.5 by 2.0-cm solid mass in the upper lobe of his right lung. Diagnosis of gastric adenocarcinoma was obtained at histological analysis by lung biopsy which revealed signet ring cells, and at clinical evaluation of his gastric symptoms (gastric pain, dyspepsia). Contemporaneously, due to haematologic pancytopenia, the patient was studied by bone marrow biopsy (BOM) that revealed a diffuse infiltration of adenocarcinoma with signet ring cells at histological analysis. The patient died of disease progression in September 2009.

The patient's sister had died of neck cancer at the age of 50 years. There was no history of malignancies in the patient's mother, father or other first-degree relatives.

## Discussion

The clinical association of florid eruption of seborrheic keratoses and diagnosis of malignancy clearly identify Leser-Trélat syndrome. Heaphy et al. [11] suggest that it would be useful to distinguish between a "sign of Leser-Trélat" and a "syndrome of Leser-Trélat." They propose that the "sign of Leser-Trélat" be defined as a sudden acute efflorescence of seborrheic keratoses sometimes accompanied by pruritus or acanthosis nigricans (or both). According to this definition, the sign may be present with or without occult malignancy and is detectable on history and physical examination alone. The term "syndrome of Leser-Trélat" would then be used to describe a paraneoplastic syndrome in patients with the "sign of Leser-Trélat" in whom an occult malignancy was identified after the appearance of the sign.

Our report appears to demonstrate that, even when the sign of Leser-Trélat is confirmed by the diagnosis of anamnestic or recent malignancies, patients affected by this syndromic disorder should be carefully monitored for different visceral and potentially multiple primitive malignancies. This instrumental surveillance strategy should be tailored taking into account either the novel sudden eruption of SK or the size and change in number of known SK. Regarding the type of associated malignancies, the sign of Leser-Trélat is usually associated with an adenocarcinoma, often in the stomach or colon, although squamous cell carcinoma, lymphoma, and leukaemias may also be observed. However, about 20% of patients reportedly have a lymphoma or a leukaemia rather than an adenocarcinoma. The distribution of the underlying cancer is similar to that of malignant acanthosis nigricans, that is, adenocarcinomas predominate, especially in the stomach and the colon.

Seborrheic keratoses are exceedingly common and various authors have doubted the existence of anything other than a chance or coincidental association in the sign of Leser-Trélat [11]. In other known cutaneous paraneoplastic syndromes, the rarity of the skin manifestation also permits a specific correlation to the origin of the underlying malignancy (Table 1).

There is some evidence for a humoral link between various malignancies and the rapid onset of paraneoplastic syndrome. Ellis et al. [10] identified a role for growth factors in the production of cutaneous paraneoplastic syndromes. Normally, epidermal growth factor (EGF) receptors are present on basal keratinocytes and decrease as the keratinocytes differentiate in the upper epidermal layers. In the case presented, the immunohistochemical analyses of EGFR protein revealed an intense membranous staining in the basal keratinocytes and in all the upper epidermal layers except the stratum corneum; in addition, cytoplasmic positivity was revealed in almost 50% of the cells (Figure 2). This is in accordance with the data of Ellis et al. [10], in which biopsy specimens of acanthosis nigricans, seborrheic keratoses, and acrochordons showed intense staining for EGF receptors in all epidermal layers, except the stratum corneum, showing that EGFR overexpression may be one of the concomitant pathognomonic mechanisms responsible for sudden multiple eruption of SK.

Importantly, a question to consider is whether, given the relative frequency of such lesions, routine immunohistochemical (IHC) testing may be warranted for any multiple SK. We propose that EGFR immunohistochemical analysis indicates the presence of LTS only when some additional clinical information is present (sudden multiple SK eruption, acute morphological changes of chronic multiple SK, association with acanthosis nigricans or other paraneoplastic skin manifestations, early age at onset of SK, individual or familial history of malignancies). In these settings, the SK sign should be carefully examined through IHC tests even when they appear in individuals without evidence of visceral malignancy. Systematic analyses of EGFR IHC expression in multiple SK of patients affected by LTS will provide more information on the role of concomitant hormonal alterations underlying multiple SK and visceral cancerogenesis.

In the literature, only a few cases of LTS have been reported [8,9,12]. In fact, although seborrheic keratoses are common findings, the early appearance of these keratoses and their eruptive nature are uncommon. However, we believe that LTS is underestimated probably because of the lack of importance assigned to multiple SK (mSK) in personal clinical surveys. As multiple SK may be under- or misdiagnosed in common clinical practice, we suggest that a proper dermatologic evaluation should be conducted in all cases of sudden eruption or intensification of chronic SK in order to fully evaluate the frequency of this association.

Patients with sign of Leser-Trélat should undergo a diagnostic screening programme for malignant disease along with patients with known Leser-Trélat syndrome who present with a recent acute and florid eruption of their seborrheic keratoses. We propose the importance of combining the molecular features of multiple seborrheic keratoses with EGFR immunohistochemistry analyses to determine the likelihood of Leser-Trélat syndrome and consequent high risk of visceral cancer development.

## Consent

Written informed consent was obtained from the patient for publication of this case report and accompanying images. A copy of the written consent is available for review by the Editor-in-Chief of this journal.

## Competing interests

The authors declare that they have no competing interests.

## Authors' contributions

GP and GL were responsible for the clinical management of the patient, acquisition of data, and drafting the manuscript; LL was responsible for histologic diagnosis and EGFR immunohistochemical analysis and discussion; AG was involved in discussion of the manuscript; SS was supervisor of clinical management of the patient and interpretation of the data. All authors read and approved the final manuscript.
